# Fish pathogen binding to mucins from Atlantic salmon and Arctic char differs in avidity and specificity and is modulated by fluid velocity

**DOI:** 10.1371/journal.pone.0215583

**Published:** 2019-05-24

**Authors:** János Tamás Padra, Abarna V. M. Murugan, Kristina Sundell, Henrik Sundh, John Benktander, Sara K. Lindén

**Affiliations:** 1 Department of Medical Chemistry and Cell Biology, University of Gothenburg, Gothenburg, Sweden; 2 Department of Biological and Environmental Sciences, University of Gothenburg, Gothenburg, Sweden; Xiamen University, CHINA

## Abstract

Disease outbreaks are limiting factors for an ethical and economically sustainable aquaculture industry. The first point of contact between a pathogen and a host occurs in the mucus, which covers the epithelial surfaces of the skin, gills and gastrointestinal tract. Increased knowledge on host-pathogen interactions at these primary barriers may contribute to development of disease prevention strategies. The mucus layer is built of highly glycosylated mucins, and mucin glycosylation differs between these epithelial sites. We have previously shown that *A*. *salmonicida* binds to Atlantic salmon mucins. Here we demonstrate binding of four additional bacteria, *A*. *hydrophila*, *V*. *harveyi*, *M*. *viscosa* and *Y*. *ruckeri*, to mucins from Atlantic salmon and Arctic char. No specific binding could be observed for *V*. *salmonicida* to any of the mucin groups. Mucin binding avidity was highest for *A*. *hydrophila* and *A*. *salmonicida*, followed by *V*. *harveyi*, *M*. *viscosa* and *Y*. *ruckeri* in decreasing order. Four of the pathogens showed highest binding to either gills or intestinal mucins, whereas none of the pathogens had preference for binding to skin mucins. Fluid velocity enhanced binding of intestinal mucins to *A*. *hydrophila* and *A*. *salmonicida* at 1.5 and 2 cm/s, whereas a velocity of 2 cm/s for skin mucins increased binding of *A*. *salmonicida* and decreased binding of *A*. *hydrophila*. Binding avidity, specificity and the effect of fluid velocity on binding thus differ between salmonid pathogens and with mucin origin. The results are in line with a model where the short skin mucin glycans contribute to contact with pathogens whereas pathogen binding to mucins with complex glycans aid the removal of pathogens from internal epithelial surfaces.

## Introduction

In fish aquaculture, disease outbreaks limit the development of the industry because of associated ethical and economic issues. Current remedies by vaccination and/or antibiotic treatment are far from ideal: vaccines do not provide the ultimate solution to the problem due to severe side effects and limited efficacy for some pathogens [1]. Use of antibiotics adds to the spreading of antibiotic resistant bacteria and genes into our environment [2]. To increase fish welfare and aquaculture productivity, an improved understanding of host-pathogen interactions is necessary.

For infection to occur, bacteria have to contact the host’s epithelial surfaces, mainly constituted by the skin, gills and the gastrointestinal tract. These organs are covered by a mucus layer built of highly glycosylated gel-forming mucins [3]. Atlantic salmon skin mucins are less glycosylated and carry a lower diversity of structures than intestinal mucins, and linear structures dominate the skin glycan repertoire whereas intestinal mucin *O*-glycans are predominantly branched [4, 5]. Atlantic salmon intestinal mucins provide more adhesion sites for the pathogenic bacterium *Aeromonas salmonicida* than skin mucins [4]. That mucin glycans trap pathogens on human gastric mucins will likely benefit the host as the pathogens are kept closer to the lumen center and away from the epithelial surface, minimizing host-pathogen interactions at the epithelial surfaces [6, 7]. This concept potentially applies to fish gastrointestinal mucins too. On the other hand, for tissues like the skin and gills exposed to a fluid velocity of the surrounding water, it can be hypothesized that weak mucin-pathogen interactions are beneficial as the pathogens would be washed off by the water flow thereby minimizing pathogen attachment [4, 8].

We have previously demonstrated that *A*. *salmonicida* binds to Atlantic salmon mucin *O*-glycans terminating in *N*-acetylneuraminic acid (NeuAc) [4]. The glycan structures involved in attachment for most other fish pathogenic bacteria are unknown. Among the pathogens studied here, *Aeromonas hydrophila* is a Gram-negative pathogen causing motile Aeromonas septicemia (MAS) in freshwater fish [9]. It has a wide host-range, infecting all classes of vertebrates [10–13], including humans [14]. *A*. *hydrophila* has zoonotic importance as it can cause septicemia, necrotizing fasciitis, gas gangrene and meningitis, mainly in immunocompromised patients [15–17]. *Aeromonas hydrophila* frequently infects rainbow trout [18–20] and has been detected attached to the distal intestinal wall of asymptomatic Arctic char and Atlantic salmon [21]. *A*. *hydrophila* is an opportunistic pathogen and whether it is autochthonous or allochthonous to the microbiota of salmonids is debated [21]. *A*. *hydrophila* has at least two (98 kDa and 150 kDa) cell surface proteins that bind to bovine submaxillary mucin, crude salmon skin mucus and porcine gastric mucin [22].

*Vibrio harveyi* (also called *V*. *carchariae*) is a luminescent, Gram-negative marine bacterium that infects crustaceans [23–25], mollusks [26–28] and fish [29–31] including Atlantic salmon [32]. Infection in Arctic char has not been described to date.

*Moritella viscosa* is the causative agent of winter ulcer disease, causing mortality and financial loss in on-growing salmonids reared in sea-cages below 10 °C [33]. In Atlantic salmon, *M*. *viscosa* infection has been described as a systemic disease, characterized by skin ulcers, gill pallor, fin rot, muscle damage and hemorrhages in liver and pyloric ceca [34–37] in both vaccinated and unvaccinated fish [38].

*Yersinia ruckeri* is a Gram-negative rod-shaped enterobacterium that causes enteric red mouth disease (ERM) and infects wild and farmed salmonids starting with low level mortalities that may result in high cumulative stock losses in farms [39, 40]. Histological examination of rainbow trout experimentally infected with *Y*. *ruckeri* indicated that gills are a portal of entry after which it spreads to other organs [41]. *Y*. *ruckeri* adhered more avidly to gill and gut explants of rainbow trout than *E*. *coli* [42]. The structure and expression of putative adhesins have been described in *Y*. *ruckeri* [43] but the function of these adhesins is not fully understood.

*Vibrio salmonicida* is the causative agent of Cold-water vibriosis (CV), a septicemic condition of mainly farmed Atlantic salmon but identified in other salmonids as well [44]. The gills, skin and GI tract have previously been suggested as the portal of entry in *V*. *salmonicida* infections, but no clear conclusion has been made [45–48]. Bjelland *et al*. recently described the pathomechanism of CV in Atlantic salmon with bacteria appearing first in the blood, intestines, mouth and gills, but not on the skin surface, shortly after an immersion challenge [49]. Later on *V*. *salmonicida* persisted in the intestine and could be found with increasing number in the skin, liver and head kidney [49].

The global demand for Atlantic salmon, *Salmo salar* L., as food for human consumption is increasing worldwide and the farming industry is under steady expansion. In Sweden and the Nordic countries, the cold water salmonid species Arctic charr, *Salvelinus alpinus*, is also of commercial importance. Iceland, Sweden and Norway are the main producers and together supply more than 90% of the European market [50, 51].

The objectives of this study were to identify if salmonid bacterial pathogens (*A*. *hydrophila*, *V*. *harveyi*, *Y*. *ruckeri*, *M*. *viscosa* and *V*. *salmonicida*) bound to salmonid mucins from different tissues and if the binding differed in specificity and avidity. Furthermore, we investigated if fluid velocity affected the bacterial binding, and if that effect was different regarding the short skin glycans in comparison to the more complex branched intestinal glycans.

## Materials and methods

### Fish and sampling procedure

Atlantic salmon parr (Långhult lax, Långhult, Sweden) were transported to the Department of Biological and Environmental Sciences and kept in a FW recirculating aquaculture system (RAS) with 500 liter tanks supplied with 10°C FW, supplemented with 10% seawater (yielding a salinity of 2–3‰), at a flow rate of 8.5 l/min. The fish were exposed to a simulated natural photoperiod and were hand-fed *ad libitum* once daily with a commercial dry pellet (Nutra Olympic /3mm in diameter/, Skretting Averøy, Ltd, Stavanger, Norway).

Arctic char of the ‘Arctic superior’ breed were kept at Kälarne Research Station (Vattenbrukscentrum Norr AB, Sweden) [52] in triplicate 700 l flow-through fibreglass tanks, supplemented with 10 l/min of water at a temperature of 5.8 ± 1.8 °C.

Five fish were randomly netted, sedated in water containing metomidate (12.5 mg/l) and killed with a sharp blow to the head. Mucus from the skin was sampled by gentle scraping of the entire skin surface using microscopy glass slides. The fish were then opened longitudinally and the intestine, from the last pyloric ceca to the anus, was quickly dissected out. The intestine was cut open along the mesenteric border and the proximal region was separated from the distal at the ileorectal valve. The mucus and mucosa were scraped off using microscopy slides. The pyloric ceca and gill arches were dissected out, placed in liquid nitrogen, and pulverized using a mortar and a pestle. All samples were placed in 10 mM sodium di-hydrogen phosphate containing 0.1 mM phenylmethanesulphonyl fluoride (PMSF) at pH 6.5 (sampling buffer) to inhibit proteolytic cleavage. The experiment on Arctic char was conducted in compliance with laws and regulations concerning experiments with live animals overseen by the Swedish Board of Agriculture and approved by the Ethical Committee for Animal Experimentation in Umeå, Sweden under licence #A62-10. The experiment on Atlantic salmon was approved by the Ethical Committee for Animal Experimentation in Gothenburg, Sweden under licence #46/2009.

### Isolation and purification of mucins

The scrapings and pulverized tissues in sampling buffer were added to five sample volumes of extraction buffer (6 M GuHCl, 5 mM EDTA, 10 mM sodium phosphate buffer, pH 6.5, containing 0.1 M PMSF), homogenized with Dounce homogenizer (four strokes with a loose pestle) and stirred slowly at 4°C, overnight. The insoluble material was removed by centrifugation at 23,000 × g for 50 min at 4°C (Beckman JA-30 rotor) and the pellet was re-extracted twice with 10 ml extraction buffer. The supernatants from these three extractions were pooled and contained the GuHCl soluble mucins used in the subsequent assays. The samples were dialyzed twice against ten volumes of extraction buffer and filled up to 26 ml with extraction buffer. CsCl was added to the samples by gentle stirring and the samples were transferred to Quick Seal ultracentrifuge tubes (Beckman Coulter). The tubes were filled with 10mM NaH_2_PO_4_ to give a starting density of 1.35 g/ml and samples were subjected to density gradient centrifugation at 40,000 × g for 90h at 15°C. The fractions were collected from the bottom of the tubes with a fraction collector equipped with a drop counter. Density gradient fractions of purified mucin samples were analyzed for carbohydrates as periodate-oxidizable structures in a microtiter-based assay: Fractions diluted 1:100, 1:500 and 1:1,000 in 4M GuHCl were coated on 96-well plates (PolySorpTM, NUNC A/S, Roskilde, Denmark) and incubated overnight at 4°C. The rest of the assay was carried out at 23–24°C. After washing three times with washing solution (5mM Tris-HCl, 0.15 M NaCl, 0.05% Tween 20, 0.02% NaN_3_, pH 7.75), the carbohydrates were oxidized by adding 25 mM sodium metaperiodate in 0.1 M sodium acetate buffer, pH 5.5, for 20 min. The plates were washed again and the wells were blocked with DELFIA blocking solution (50 mM Tris-HCl, 0.15 M NaCl, 90 mM CaCl_2_, 4 mM EDTA, 0.02% NaN_3_, 0.1% BSA, pH 7.75) for 1 h. After further washing steps, the samples were incubated for 1h with 2.5 mM biotin hydrazide in 0.1 M sodium acetate buffer, pH 5.5, followed by another washing step. Europium-labeled streptavidin was diluted 1:1,000 in Delfia assay buffer (PerkinElmer; 50 mM Tris-HCl, 0.15 M NaCl, 20 mM DTPA, 0.01% Tween 20, 0.02% NaN_3_, 1.5% BSA, pH 7.75) and was added to the wells. After 1h incubation, the plates were washed six times and then incubated with Delfia enhancement solution (PerkinElmer; 0.05 M NaOH, 0.1 M phthalate, 0.1% Triton X-100, 50 mM TOPO, 15 mM b-NTA) for 5 min at 120 rpm on an orbital shaker. Fluorescence (λ_excitation_ = 340nm and λ_emission_ = 615nm) was measured using a Wallac 1420 VICTOR_2_ plate reader with the Europium label protocol (PerkinElmer, Waltham, MA, USA). Density measurements were performed using a Carlsberg pipette as a pycnometer: 300 μl of sample was aspirated into the pipette, weighed and density was calculated as g/ml. DNA concentration was calculated from light absorbance at 280 nm.

### Preparation of mucin samples

**Fig 1:**
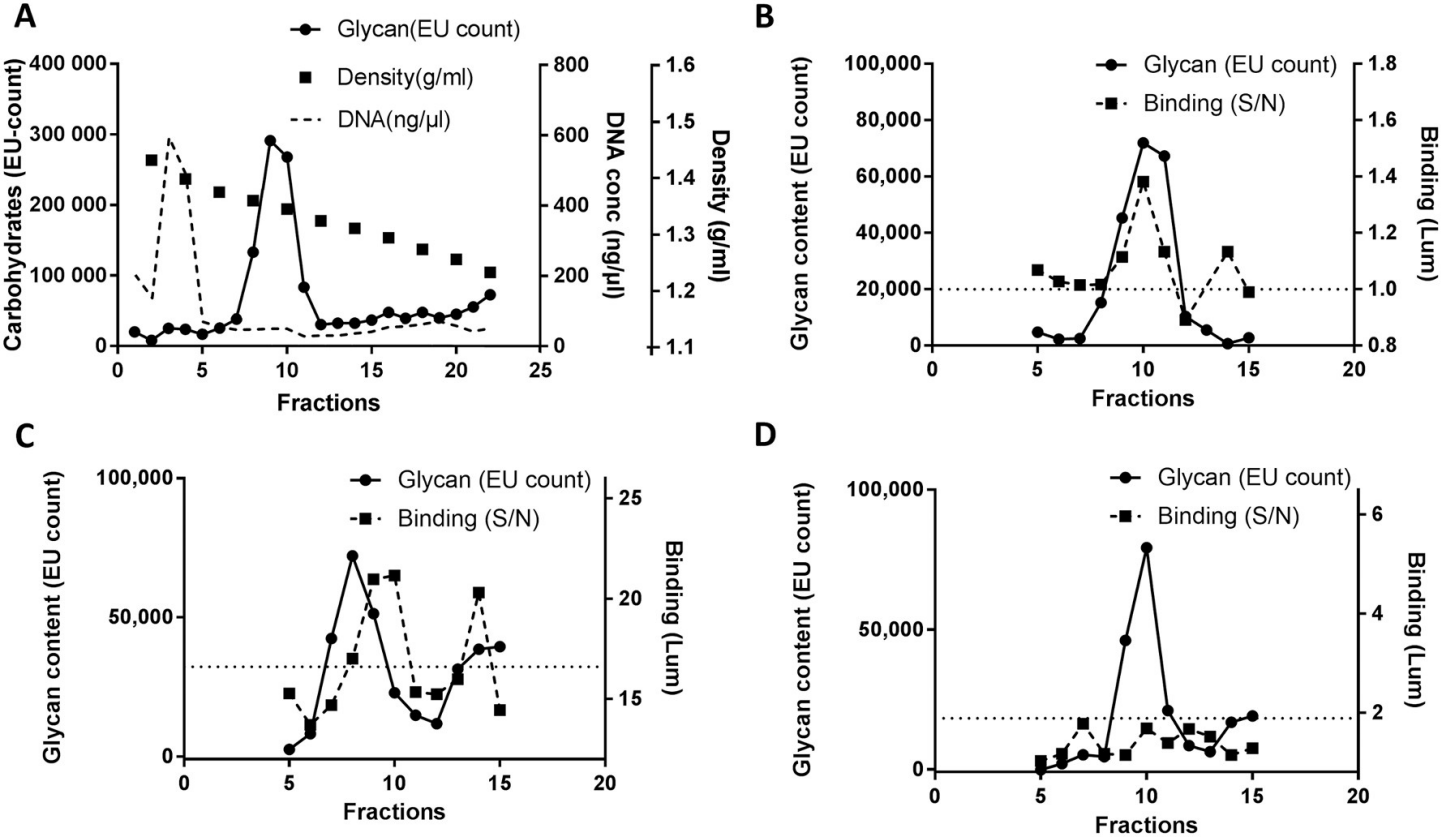
Qualitative analysis of binding specificity of pathogens to Atlantic salmon mucins. **A**. Mucins were isolated by isopycnic density gradient centrifugation, and fractions were collected from the bottom of the tube. Fractions were analyzed for carbohydrate content (glycan), DNA content and density. The glycan peak at fractions 8–11 corresponds to the mucins, and for the experiments presented in Figs 3 and 4, the fractions were pooled based on the glycan peak for each sample. **B**.-**D**. Examples of pathogen binding patterns to gradient fractions. **B**. Low avidity, mucin-specific binding of *M*. *viscosa* to an Atlantic salmon skin sample. The binding signal is low but exceeds that of the control and follows the mucin glycan peak (fractions 9–11 in this sample). **C**. High avidity, mucin-specific binding of *A*. *hydrophila* to Atlantic salmon gill sample. The binding follows the mucin glycan peak (fractions 7–10), although avidity appears stronger to a low density glycoform of the main mucin peak (i.e. the binding curve is shifted slightly to the right of the main mucin peak). **D**. Absence of *Y*. *ruckeri* binding to a pyloric cecal sample. The binding signal to the sample is lower than to the non-mucin control and does not follow the mucin glycan peak (fractions 9–11). Binding is expressed as signal/noise luminescence. The horizontal dashed lines denote the binding signal of bacteria to the plastic well. The results of all these qualitative binding analyses are summarized in Table 1. Abbreviations: EU count = europium count; Lum = luminescence, expressed as signal/noise (S/N).

Gradient fractions containing mucins were pooled together to obtain one sample from each fish and epithelial site (e.g. fractions 9–11 in Fig 1A). Mucin concentrations in pooled samples were determined based on their carbohydrate content: serial dilutions of the samples were compared with a standard curve prepared from a fusion protein, constructed from MUC1, 16TR and IgG2a Fc. The standard curve started at a concentration of 20 mg/ml and followed by seven 1:2 serial dilutions. The carbohydrates were detected as periodate-oxidizable structures in a microtiter-based assay as described above. This method of concentration determination was chosen as all mucins do not come into solution after freeze drying, and determining concentration by freeze drying therefore can contain large errors as well as selectively remove certain mucin species. Since bacterial-mucin interactions largely occur via the mucin glycans [3], this method ensures the glycan content is the same in all the samples.

### Bacterial culture conditions

*A*. *hydrophila* (ATCC 7966) and *Yersinia ruckeri* (ATCC 29473) were cultured on Nutrient agar (Difco) at 26°C. *V*. *harveyi* BB170 (courtesy of Bonnie Bassler) was cultured on Marine agar (Difco) at 26°C. *M*. *viscosa* (ATCC BAA-105) and *V*. *salmonicida* (ATCC 43839) were cultured on Tryptic Soy Agar (Oxoid) at 15°C. *A*. *salmonicida subsp*. *salmonicida* strain VI-88/09/03175 (culture collection, Central Veterinary Laboratory, Oslo, Norway) was cultured on Tryptic Soy Agar (Oxoid) at 18°C. All bacterial strains were stored in the corresponding broth medium with 15% glycerol at -80°C.

### Bacterial binding assays

Samples were diluted in 4M GuHCl/PBS and coated on white opaque plates (Costar) for binding analysis. Plates were incubated at 4°C overnight and then washed with Phosphate Buffered Saline (PBS, 140 mM NaCl, 2.7 mM KCl, 10 mM phosphate buffer, pH 7.4) + 0.05% Tween (PBS-T) three times. Plates were blocked with 200μl 0.5% Bovine Serum Albumine (BSA; Sigma-Aldrich) in PBS-T (Blocking buffer) for 1 hour at 4°C. Blocking buffer was discarded from the plate. Bacteria cultured in the log phase of growth were washed three times at 3,000 × g for 5 min and resuspended in blocking buffer (pH 7.8) to OD_600_ = 0.1 diluted 1:20 and 100μl was added to the wells. Plates were incubated for 2h at 10°C. This temperature inhibits or slows down growth of the bacteria, but all studied species stay alive. Wells were washed with chilled PBS-T three times. 100μl Bac Titer-Glo^™^ reagent and 100μl freshly prepared PBS (both equilibrated at RT) were added to the wells and incubated for 10 min to achieve equal heat distribution in the plate. Total luminescence was read in a Clariostar plate reader (Berthold technologies).

#### Qualitative binding analysis

Fractions from the whole density gradient from 1–2 fish from each epithelial site were coated onto the plates. Whole gradients include both mucin and non-mucin molecules enabling the test for mucin-specific binding. The bacterial binding curves were evaluated in parallel with curves for DNA, mucins and non-mucin proteins/glycoproteins. Relative luminesce values forming a distinct peak coinciding with mucin containing fractions were deemed specific and the binding avidity was scored based on the peak height of the relative luminescence values (marked +, ++ or +++). If the relative luminesce values of the fractions containing mucins did not exceed those of the baseline and non-mucin controls (binding avidity of bacteria to plastic well only) and/or the peak did not coincide with the mucin containing carbohydrate peak, we concluded lack of mucin specific binding (marked -).

#### Quantitative binding analysis

The mucin containing fractions from the gradients were pooled into one mucin sample for each individual and tissue (see Fig 1A for an example of how the fractions were pooled). Mucins were diluted in 4M GuHCl/PBS before coating the plates. The binding was analyzed at four mucin concentrations (in 2-fold dilution steps) with results that were similar when the binding signal was expressed per unit of carbohydrate signal for at least three of the concentrations (i.e., the assay was performed within a linear range), including a glycan value of 20,000 europium counts (corresponding to approximately 4 μg/ml mucin). For each analysis, parallel microtiter plates (Nunc PolySorp) were coated for glycan detection analysis to ensure that small differences in binding were not due to mucin coating differences. Plates were incubated at 4°C overnight and then washed with Phosphate Buffered Saline (PBS, 140 mM NaCl, 2.7 mM KCl, 10 mM phosphate buffer, pH 7.4) + 0.05% Tween (PBS-T) three times. The relative luminescence values were transformed into CFU values using a standard curve. The standard curves were produced by making a serial dilution of each bacterial species (starting at OD_600_ = 0.1; dilution factor = 10; number of dilutions = 8; diluted in 100μl PBS). The bacterial suspensions were mixed with Bac Titer-Glo and read as described above. Linear regression of CFU counts against relative luminescence was used to transform mucin binding data to CFU counts. This makes the binding assays suitable to enumerate bound bacteria for inter-species and inter-tissue comparisons. The background signal (signal from wells with no mucin coating) was subtracted before transforming the luminescence signal into CFU equivalents based on the standard curves for each pathogen.

### Binding assays with fluid velocity

Fluid velocity in binding assays (described above) was generated by orbital shaking of the 96-well plates during the 2h incubation step with bacteria at RT. Bacteria were suspended in Bold Modified Basal Freshwater Nutrient Solution (Sigma Aldrich) containing 0.5% Bovine Serum Albumin (Sigma Aldrich). RPM of the orbital shaking was converted to linear velocity using the following formula: v = f × 2π × r, where “v” is linear velocity, “f” is frequency in round/s, and “r” is radius of the plate well.

### Statistical analyses

Statistical analyses were performed using the Graph Pad Prism 7.0 (GraphPad Software Inc.) software package. Normality and homoscedasticity of data were investigated using the Kolmogorov-Smirnov test and Bartlett’s test, respectively. The hypothesis that the binding avidity of pathogens differed between mucins of different tissue origin was tested by Kruskal-Wallis test followed by Dunn´s Post Hoc test. The hypothesis that fluid velocity had an effect on bacterial binding to mucins was analyzed by One-Way ANOVA followed by Dunnett′s multiple comparisons test with the static condition used as control. The level of significance was set at p≤0.05.

## Results

### Binding specificity to mucins from different epithelial sites of Atlantic salmon and arctic char varies between pathogens

Mucins were purified using isopycnic density gradient centrifugation, which separated the mucins (fractions 8–11, Fig 1A) from DNA (higher density, fractions 3–4, Fig 1A) and from less glycosylated proteins and lipids (lower density, fractions 15–23, Fig 1A). Baseline separation between the peaks containing mucins and DNA ensured high mucin quality for subsequent analyses. To qualitatively analyze the binding specificity of pathogens used in this study, we carried out binding assays on the gradient fractions. The binding was considered mucin-specific if the binding curve coincided with the glycan curve, representing the mucins (Fig 1B and 1C). This includes also pathogen binding patterns that are slightly shifted from the main glycan peak (Fig 1C), as the heterogeneous nature of mucins leads to that mucin glycoforms can have slightly different density than the main mucin population and pathogen affinity can be higher to certain glycoforms [53, 54]. Between the two examples (Fig 1B and 1C), we considered the binding of *M*. *viscosa* to Atlantic salmon skin sample to be specific but of low avidity, as the signal exceeded the background signal only by 40% (Fig 1B). On the other hand, *A*. *hydrophila* binding to Atlantic salmon gill mucins was both specific and of high avidity, exceeding the background luminescence 20-fold in the fractions containing the mucins (Fig 1C). When the binding avidity of bacteria to the plastic well was similar or higher than biding to the mucin containing fractions or the binding pattern differed from that of the glycan peaks, we deemed the binding unspecific. This was e.g. the case with *Y*. *ruckeri* binding to the pyloric cecal mucins (Fig 1D).

*A*. *hydrophila*, *V*. *harveyi* and *M*. *viscosa* specifically bound to mucins derived from all studied Atlantic salmon and arctic char epithelial sites (Table 1). Among the pathogens, *A*. *hydrophila* showed the highest binding avidity to mucins, *V*. *harveyi* avidity was intermediate whereas the avidity of the remainder of the pathogens was relatively low (Table 1). *Y*. *ruckeri* bound to all investigated mucins except pyloric cecal mucins (Table 1). No specific binding to mucins was detected with *V*. *salmonicida* (Table 1). Therefore, *V*. *salmonicida* was not included in the quantitative analysis of bacterial binding to pooled mucins. The pathogen adhesion level was similar to intestinal mucins from Atlantic salmon and Arctic char.

**Table 1:** Presence or absence of mucin specific binding of pathogens.

Bacterial species	Mucin type		Specific Binding
***Aeromonas hydrophila***	Atlantic salmon	Skin	+++
		Gill	+++
		Pyloric Ceca	+++
		Proximal Intestine	+++
		Distal Intestine	+++
	Arctic char	Distal Intestine	+++
***Vibrio harveyi***	Atlantic salmon	Skin	++
		Gill	++
		Pyloric Ceca	++
		Proximal Intestine	++
		Distal Intestine	++
	Arctic char	Distal Intestine	+
***Moritella viscosa***	Atlantic salmon	Skin	+
		Gill	+
		Pyloric Ceca	+
		Proximal Intestine	+
		Distal Intestine	+
	Arctic char	Distal Intestine	+
***Yersinia ruckeri***	Atlantic salmon	Skin	+
		Gill	+
		Pyloric Ceca	-
		Proximal Intestine	+
		Distal Intestine	+
	Arctic char	Distal Intestine	+
***Vibrio salmonicida***	Atlantic salmon	Skin	-
		Gill	-
		Pyloric Ceca	-
		Proximal Intestine	-
		Distal Intestine	-
	Arctic char	Distal Intestine	-

Based on the qualitative evaluation of glycan vs. binding curve analysis (e.g. Fig 1), pathogens showed specific binding (+-+++) or absence of specific binding (-) to mucins from different epithelial locations/fish species. The former means that the binding was higher to mucin containing fractions compared to non-mucin fractions while the latter means that the binding of bacteria to mucins did not exceed markedly the binding to non-mucin fractions of the gradient. The number of “+” indicates the amplitude of the binding signal.

### Mucin binding avidity and specificity differ between pathogens

**Fig 2:**
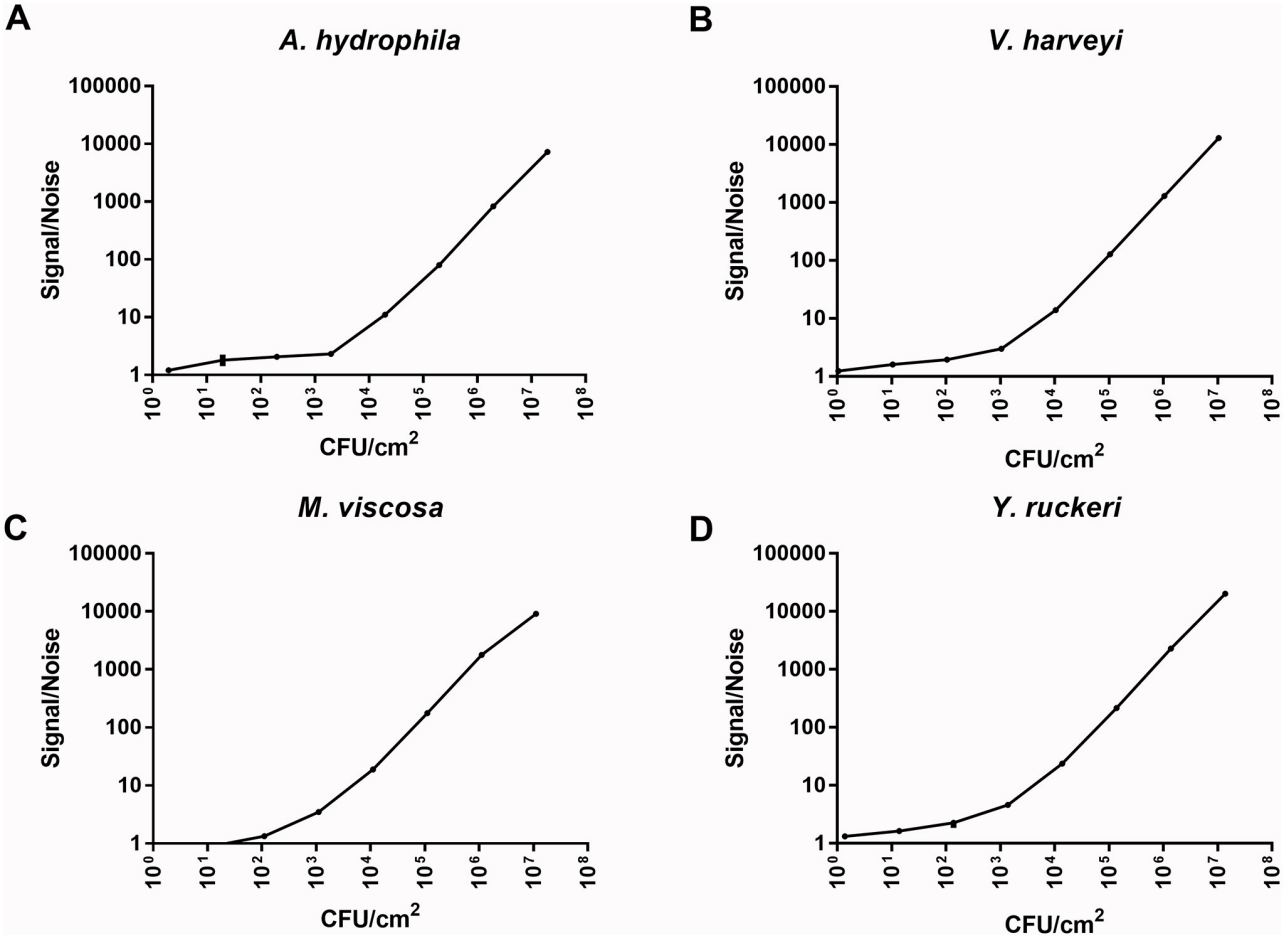
Standard curves for calculation of adherent bacteria from luminescent signals. Luminescence produced by *A*. *hydrophila* (A), *V*. *harveyi* (B), *M*. *viscosa* (C) and *Y*. *ruckeri* (D) using the Bac Titer-Glo^™^ reagent. The standard curves allow for transformation of Signal/noise ratios of luminescent signals into number of adhered bacteria/cm^2^ surface of the plate well using linear regression. The data points are expressed as mean±SEM and are technical replicates (due to low variation between replicates, the error bars are small, and what looks like symbols in the graph are the error bars). CFU = colony forming unit.

**Fig 3:**
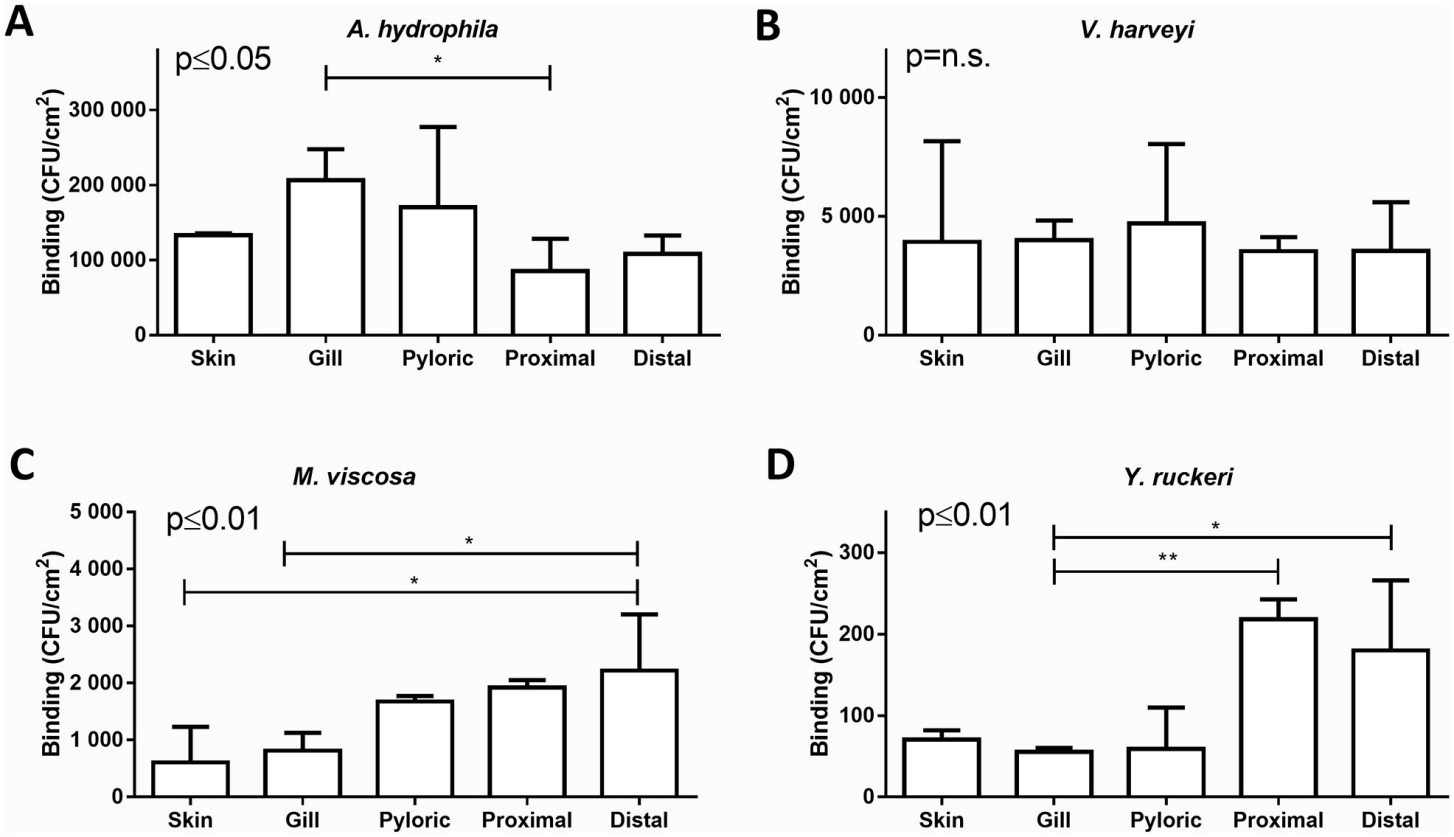
Quantitative analysis of pathogen binding to Atlantic salmon mucins. Mucin containing fractions from individual fish and tissue sites were pooled according to the method shown in Fig 1A (n = 5 for each tissue). Pathogen binding to each of these 25 samples was analyzed using the Bac Titer-Glo method, and the luminescence signals were transformed to CFU/cm^2^ according to standard curves for each pathogen (Fig 2) to allow comparison of binding levels between pathogens. **A**. *A*. *hydrophila* binding to gill mucins was higher compared to the proximal intestinal mucins: p≤0.05; n = 5). **B**. *V*. *harveyi* bound with no distinguishable organ preference (p = n.s.). **C**. The level of *M*. *viscosa* binding differed between mucin groups (distal intestine vs. skin and gill: p≤0.05). **D**. *Y*. *ruckeri* bound to proximal and distal intestinal mucins more than to gill mucins (p≤0.01 and p≤0.05). Bars denote median ± interquartile range of biological replicates, after subtracting the background signal. The results were reproduced twice. Statistics: Kruskal-Wallis test by ranks with Dunn´s Post Hoc test to compare binding to mucins from different epithelial sites. The numerical p values on the graphs show the result of the test, without the post hoc test. Abbreviations: Pyloric = pyloric cecal mucins; Proximal = proximal intestinal mucins; Distal = distal intestinal mucins.

We investigated binding of *A*. *hydrophila*, *V*. *harveyi*, *M*. *viscosa* and *Y*. *ruckeri* to mucins isolated from skin, gills, pyloric ceca, proximal and distal intestine from five Atlantic salmon. To be able to compare the level of binding between the bacterial species, standard curves were made for each pathogen (Fig 2), and luminescence readouts from the assays were transformed to CFU/cm^2^ (Fig 3). The fractions containing mucins were pooled into one mucin sample per individual and epithelial site.

Atlantic salmon mucins bound 25 to 1000-fold (depending on epithelial site and pathogen) more *A*. *hydrophila* (~85k to ~206k CFU/cm^2^) than other investigated pathogens (Fig 3A). In decreasing order of bacteria attached to the mucins, *V*. *harveyi* (~3,500 to ~4,700 CFU/ cm^2^, Fig 2B), *M*. *viscosa* (~600 to ~2,200 CFU/cm^2^, Fig 3C) and *Y*. *ruckeri* (~60 to ~220 CFU/cm^2^, Fig 3E) followed.

*A*. *hydrophila* had higher avidity to gill mucins compared to proximal intestinal mucins (>2-fold difference, p≤0.05; n = 5, Fig 3A). *V*. *harveyi* binding was equally strong in all tissues (p = 0.85; n = 5, Fig 3B). Mucins originating from the distal intestine bound 3-fold more *M*. *viscosa* compared to mucins from skin and gills (p≤0.05; n = 5, Fig 3C). More *Y*. *ruckeri* bound to proximal and distal intestinal mucins compared to gill mucins (p≤0.01 and p≤0.05, respectively; n = 5, Fig 3D).

### Binding between Atlantic salmon mucins and pathogens is modulated by external fluid velocity

**Fig 4:**
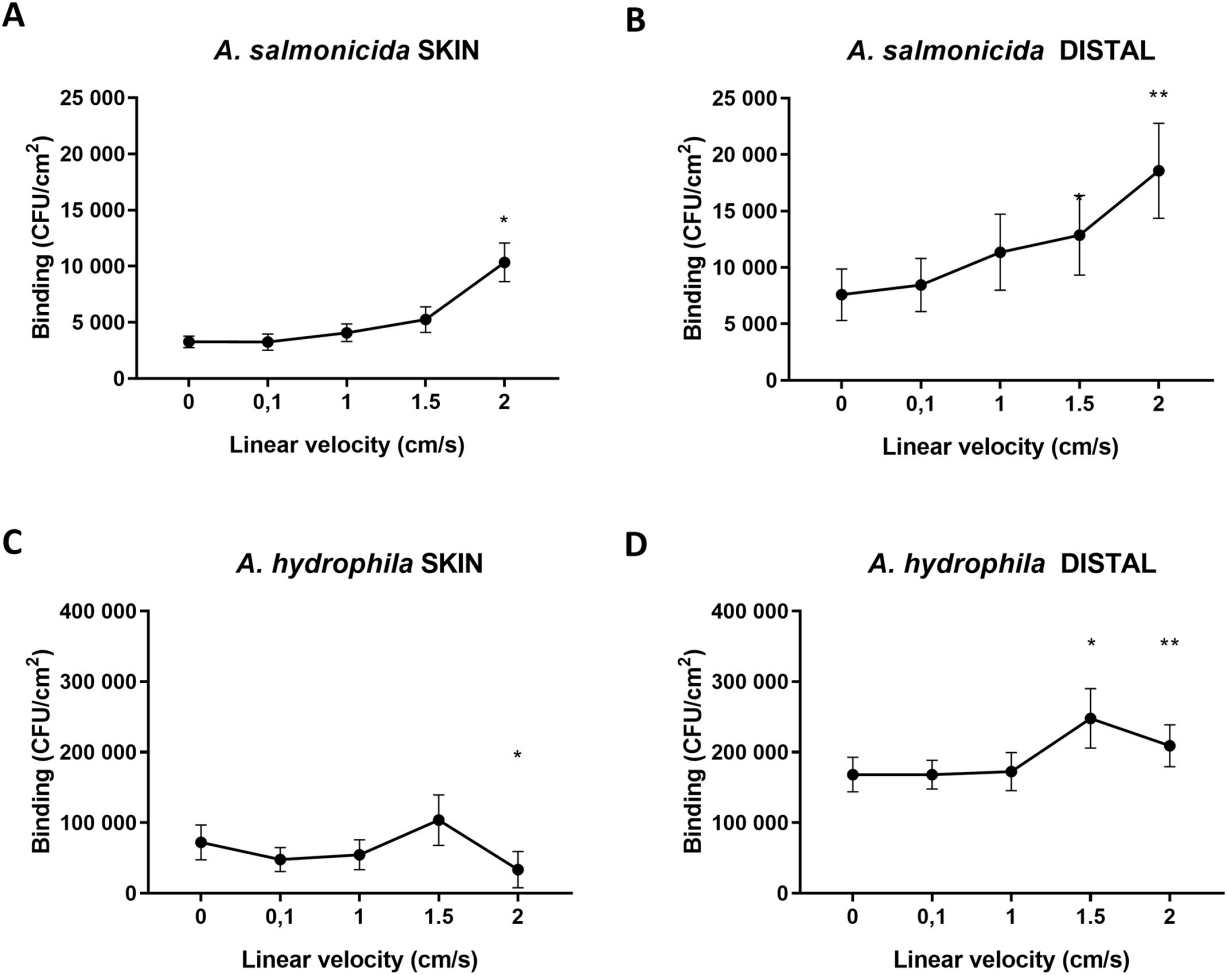
Effect of fluid velocity on bacterial binding to mucins. **A and B**. *A*. *salmonicida* binding to Atlantic salmon skin and distal intestinal mucins increased with growing linear velocity of the surrounding liquid. *A*. *salmonicida* bound with higher avidity to skin mucins at a fluid velocity of 2 cm/s (p≤0.05; n = 7) and to distal intestinal mucins at 1.5 cm/s and 2 cm/s fluid velocity (p≤0.05 and p≤0.01, respectively; n = 7). **C**. *A*. *hydrophila* binding to skin mucins was reduced at 2 cm/s fluid velocity (p≤0.05; n = 7). **D**. *A*. *hydrophila* binding was higher at 1.5 cm/s and 2 cm/s fluid velocity compared to the static environment (p≤0.05 and p≤0.01, respectively; n = 5). Data points represent mean±SEM of biological replicates. The results were reproduced twice. Statistics: One-Way ANOVA with Dunnet´s post-hoc test (compared to 0 cm/s velocity).

We have previously demonstrated that *A*. *salmonicida* binds to Atlantic salmon mucins [4]. *A*. *salmonicida* binding avidity to both skin and distal intestinal mucins gradually increased with increasing fluid velocities (Fig 4A and 4B, p≤0.5; n = 7). Compared to the binding levels at static conditions, a linear velocity of 2 cm/s resulted in a 3-fold increase in binding to skin mucins (p≤0.05; n = 7). Adhesion to the distal intestinal mucins increased already at 1.5 cm/s (p≤0.05) and at 2 cm/s the increase was 3-fold (Fig 4A and 4B, p≤0.01; n = 7). *A*. *hydrophila* binding to skin mucins showed a tendency to increase at 1.5 cm/s (p = 0.14, n = 7) followed by a decrease at 2 cm/s with half the amount of attached bacteria compared to a static environment (Fig 4C and 4D, p≤0.05; n = 7). *A*. *hydrophila* binding to distal intestinal mucins increased 1.5-fold at 1.5 cm/s (p≤0.05; n = 5) and 1.2-fold at 2 cm/s compared to the binding under static conditions (Fig 4C and 4D, p≤0.01; n = 5). Thus, binding to skin mucins with both pathogens is affected by fluid velocity at 2 cm/s, whereas effects on binding to distal intestinal mucins are detected already at 1.5 cm/s.

## Discussion

In this study, we demonstrated that *A*. *hydrophila*, *V*. *harveyi*, *M*. *viscosa* and *Y*. *ruckeri* bound to mucins from Atlantic salmon and Arctic char, whereas we did not detect specific binding of *V*. *salmonicida* to mucins from any of the epithelial sites. Binding specificity and avidity to mucins from different Atlantic salmon epithelial sites (skin, gills, pyloric ceca, proximal intestine and distal intestine) differed between pathogens. Mucin binding avidity was highest for *A*. *hydrophila*, then *V*. *harveyi*, *M*. *viscosa* and *Y*. *ruckeri* followed in decreasing order. *M*. *viscosa* and *Y*. *ruckeri* bound preferentially to intestinal mucins, *A*. *hydrophila* to gills and *V*. *harveyi* bound equally to mucins from all epithelial sites. Finally, we demonstrated that the effect of fluid velocity on binding to mucins differs between pathogens and mucin origin.

Among the pathogens studied here, *A*. *hydrophila* had a much higher avidity to mucins than the other pathogens (25 to 1000-fold, depending on epithelial site and pathogen). We found that higher numbers of *A*. *hydrophila* bound to gill and pyloric cecal mucins compared to mucins from other epithelia. This is in agreement with findings in channel catfish, *Ictalurus punctatus*, where the gills had the highest number of adherent bacteria peaking in the early phase post infection during a bath challenge with *A*. *hydrophila* [55]. In that experiment, the skin and intestine had lower *A*. *hydrophila* counts even if they increased over time [55], which gives further support for the gills being the primary tissue for *A*. *hydrophila* interaction. Another study in channel catfish, focusing on histological changes, showed lesions to appear first in the stomach and spleen, then in the intestine, and only after that on the gills [56]. Thus, in channel catfish, the adherence of *A*. *hydrophila* to mucins shows an inverse relationship with the pathological signs [55, 56]. This inverse relationship could be explained by the mucins potentially acting as releasable decoys, i.e. a form of host defense where bacteria are shed from the mucosal surface entrapped in mucus [6, 8].

*V*. *harveyi* can bind to red sea bream intestinal epithelial cells through the host GM4 ganglioside (NeuAcα2,3Galβ1Cer) [57, 58]. Although *V*. *harveyi* infects salmonids, it primarily infects crustaceans where it attaches to the host through its chitin binding proteins and is able to degrade chitin [59]. Conversely, nothing is known about its attachment mechanism to fish. We found that *V*. *harveyi* binds to all Atlantic salmon mucins and to arctic char distal intestinal mucins. The level of binding was similar between the tissues. The NeuAc structure is highly abundant on mucins from all epithelia investigated in this study [5]. However, the NeuAcα2,3Gal terminating structures constitute only 0–6.3% of the NeuAc containing structures, which may be a reason for the much lower binding of *V*. *harveyi* compared to *A*. *salmonicida* which binds with high avidity to Atlantic salmon mucins through NeuAc [4].

We show that *M*. *viscosa* binds to both arctic char and Atlantic salmon mucins. Winter ulcer disease is mainly characterized by localized swelling of the skin followed by ulcers. The gills have been proposed as an entry route for *M*. *viscosa* infection in Atlantic salmon, based on higher numbers of *M*. *viscosa* on gills than on skin and the intestinal tract after bath challenge [60]. Our data show that the binding of *M*. *viscosa* to mucins from Atlantic salmon skin and gill was lower than binding to mucins from the intestinal tract. Among the intestinal epithelial sites, binding was most pronounced to distal intestinal mucins. The lower number of bacteria in the intestinal sites of Atlantic salmon after bath challenge [60], despite that the avidity of these bacteria to intestinal mucins was higher than to other sites as reported in the current study, may potentially be explained by higher level of bacterial clearance through the entrapment of bacteria and shedding of intestinal mucins [6, 8].

Data from a rainbow trout immersion infection model, suggest that *Y*. *ruckeri* infection may start in the gills [41]. Tobback et al. demonstrated that *Y*. *ruckeri* bind to rainbow trout gill mucosal scrapings to a higher extent than to intestinal mucosal scrapings [61]. These mucus scrapings were mixed with PBS and centrifuged to remove cell debris. In our experiments, where we used purified mucins, *Y*. *ruckeri* bound more avidly to intestinal mucins than to skin and gill mucins. These results may appear to be contrasting, however mucosal scrapings contain a range of small proteins that coat faster to microtitre plates than mucins and thereby is likely to have a proportionally higher presence on the plastic surface compared to the large, slow coating mucins when small molecules are present in the coating process. Therefore, combining Tobback et al.’s data with ours do not demonstrate that there is a difference between *Y*. *ruckeri* adhesion to rainbow trout and Atlantic salmon mucins, although the possibility cannot be excluded. Tobback et al. also demonstrated that binding of *Y*. *ruckeri* to mucosal scrapings was partially inhibited by galactose [61]. Galactose terminating structures are present on Atlantic salmon mucin glycans [5, 62], and could thus be a factor in the interaction between mucins and *Y*. *ruckeri*.

We did not find any specific binding of *V*. *salmonicida* to Atlantic salmon or Arctic char mucins, suggesting that if mucins are a part of the Atlantic salmon defense against this pathogen, it is through steric hindrance and not through a decoy binding mechanism [8]. However expression of bacterial proteins involved in motility, oxidative defense, and general stress responses change after subjecting *V*. *salmonicida* to non-mucin molecules from mucus [63], indicating that *V*. *salmonicida* may interact with other molecules than mucins present in mucus.

In this study we also analyzed the effect of fluid velocity on the adhesion of two bacterial species: *A*. *salmonicida*, the binding characteristics of which we published earlier [4] and *A*. *hydrophila*. The studied fluid velocities are relevant aspects of bacterial binding to skin mucins. The fluid velocity rates studied ranged from static to moderate fluid velocities. A 2 cm/s linear velocity was the highest we could test for technical reasons, but Atlantic salmon in aquaculture can be exposed to velocities higher than this, although a speed of 1.5 × body length/s (corresponding to ~ 50cm/s here) maintained for extended periods results in reduced growth in Atlantic salmon [64]. The studied fluid velocities do not occur in the GI tract of fish as peristaltic waves in the gut of brown trout travel at a speed of 2 cm/min [65]. However, we analyzed the effect of fluid velocity on binding to distal intestinal mucins as well as we hypothesized that an advantage of skin glycans to be short and of low complexity could be to decrease the risk of pathogens attaching to the mucins while flowing past the fish surface. Indeed, none of the six pathogens studied in the current work bound with higher avidity to skin mucins than to mucins from other sites during static conditions. On the contrary, for four pathogens the binding preference was to the mucins from other epithelia, which all have more complex glycans than the skin [62]. In line with this, fluid velocity enhanced binding of *A*. *hydrophila* and *A*. *salmonicida* to intestinal mucins at 1.5 cm/s. While a tendency to an increase in adherence was observed also to skin mucins at this velocity, this was not statistically significant. At 2 cm/s, *A*. *salmonicida* adhesion was further enhanced to distal intestinal mucins and significant also to skin mucins whereas *A*. *hydrophila* binding to skin mucins decreased. We found this decrease surprising, but it was reproduced multiple independent times, and we speculate that the *A*. *hydrophila* adhesins may be more fragile than those of *A*. *salmonicida* or that the *A*. *hydrophila* binding strength to mucins is exceeded by the physical forces of the fluid, washing away part of the adherent bacteria. An important difference between these two pathogens is that *A*. *salmonicida* is aflagellar while *A*. *hydrophila* has a polar flagellum and thereby the latter might be able to actively reduce its binding by negative chemotaxis similarly to the effacing behavior of other bacterial species [66]. This behavior of active biofilm dispersal where environmental stimuli trigger changes in bacterial behavior that consequently lead to e.g. altered capsule polysaccharide qualities and later detachment has been reviewed in Aeromonads as well [67].

Infection *in vivo* requires bacterial adhesion to epithelial cells or to damaged tissue. From mammalian studies, it is known that pathogens can bind to specific carbohydrate structures present both on glycolipids and mucins, where the latter keeps the pathogen at a distance from the epithelial surface and can act as a decoy to intimate adherence to the host provided by the former [8, 68]. A balance between adherence to these different, but structurally related, molecules is likely important to the outcome of the disease [6]. Furthermore, providing a mucosal environment accommodating to a beneficial microflora may also create a niche that pathogens can utilize [62]. *A*. *hydrophila*, for which we found high avidity to mucins from all epithelial sites, can be found in the mucus of healthy Atlantic salmon as part of the microflora [69] with the possibility to develop infection when the fish become prone (e.g. in case of a louse infection [70]). After the contact is established, high mucin affinity to bacteria may help keeping pathogens entrapped in the mucus and prevent infection through immunological and mechanical defenses. Indeed, a mammalian pathogen has been shown to downregulate adhesins that bind to mucins when cultured with mucins with high binding affinity for the pathogen but not when cultured with non-binding mucins, which may be a mechanism used by the pathogen to avoid removal from the niche [71]. These complexities in mucus-pathogen interactions may be important to keep in mind when trialing different “mucus enhancing” feeds and treatments. Further knowledge in this area could be beneficial both for breeding of infection-resistant stocks and for development of therapeutic or prophylactic treatments of infection.

In conclusion, we demonstrate that avidity and specificity of binding to salmonid mucins differed among the five pathogens studied and the effect of fluid velocity on binding differed between *A*. *salmonicida* and *A*. *hydrophila*. However, in common across the six pathogens we have studied so far (including marine and freshwater opportunistic or obligate pathogens) was that none of them had a preference for skin mucins carrying short simple glycans over the more complex glycans from other epithelial sites, whereas when a preference was detected it was for the mucins with complex glycosylation. Fluid velocity can modulate the mucin-bacteria interaction but the outcome, i.e increased or decreased binding, is dependent on both the bacterial species and origin of the mucins. Together, these results support the hypothesis that skin mucins are more inert to pathogen interactions than GI tract mucins, which may be an adaptation to avoid catching pathogens from the surrounding water.
